# Supplementary figures and images for: Aberrant cohesin function in Saccharomyces cerevisiae activates Mcd1 degradation to promote cell lethality
Source: PLoS Genet. 2025 Dec 10;21(12):e1011981. doi: 10.1371/journal.pgen.1011981 (PMC12711053; doi:10.1371/journal.pgen.1011981)

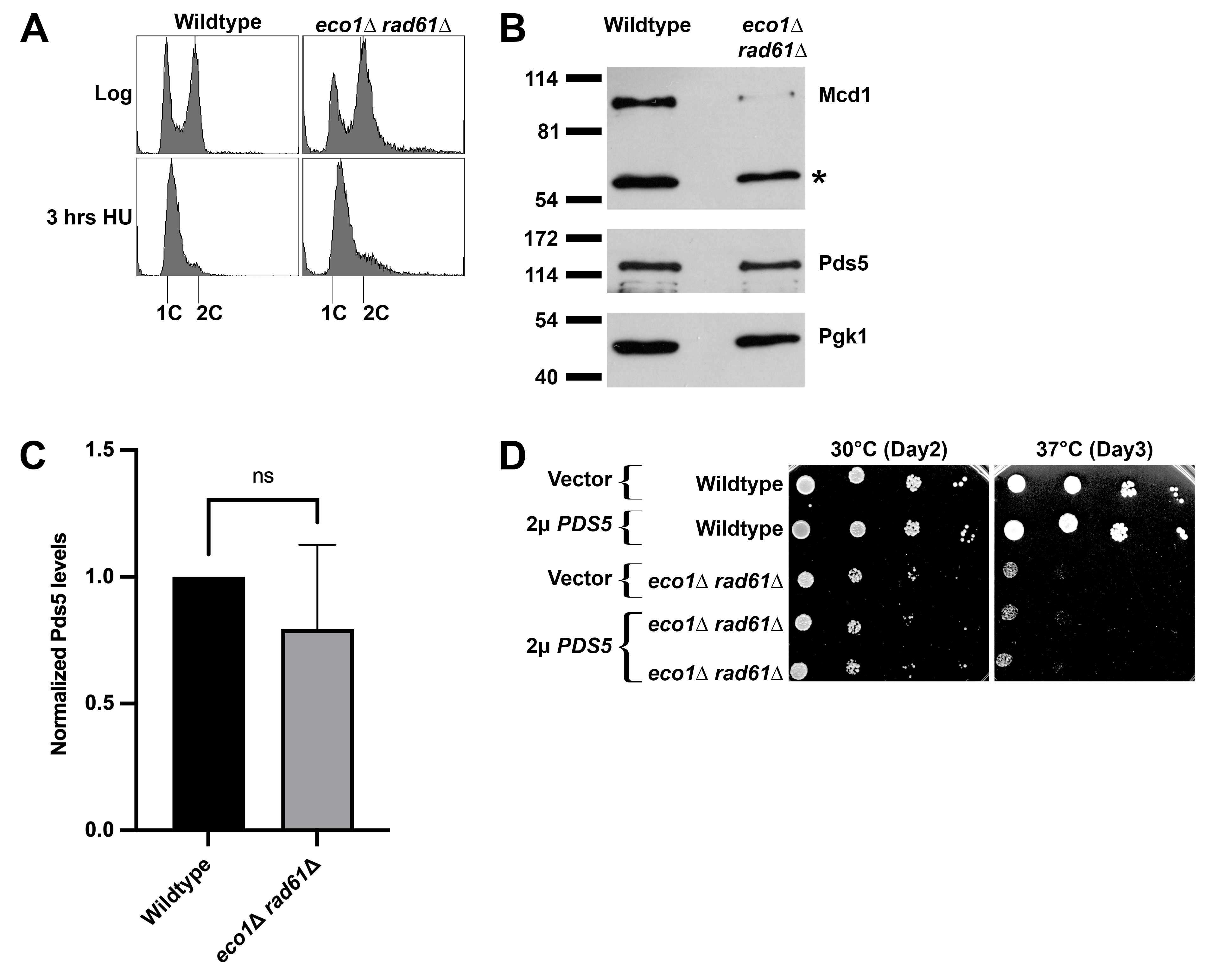

Supplement: S1 Fig — (A) Flow cytometry data of DNA content for log phase wildtype (YPH499) and eco1Δ rad61Δ (YBS828) double mutant cells arrested in S phase at 30°C for 3 hrs. (B) Representative Western Blot of Mcd1 (top panel) and Pds5 (middle) and Pgk1 (lower panel) protein obtained from extracts of HU-synchronized wildtype and eco1Δ rad61Δ mutant cells indicated in (A). * indicates non-specific band. (C) Quantification of Pds5, normalized to Pgk1 loading controls. Statistical analysis was performed using a two-tailed t-test. Statistical differences (ns) are based on a P > 0.05 obtained across three experiments (n = 3). Error bars indicate the standard deviation. (D) Growth of 10-fold serial dilutions of wildtype cells overexpressing vector alone (YBS4067) or overexpressing PDS5 (YBS4071) and eco1Δ rad61Δ overexpressing vector alone (YBS4069) or overexpressing PDS5 (YBS4075, YBS4076). (TIF) [file pgen.1011981.s001.tif]

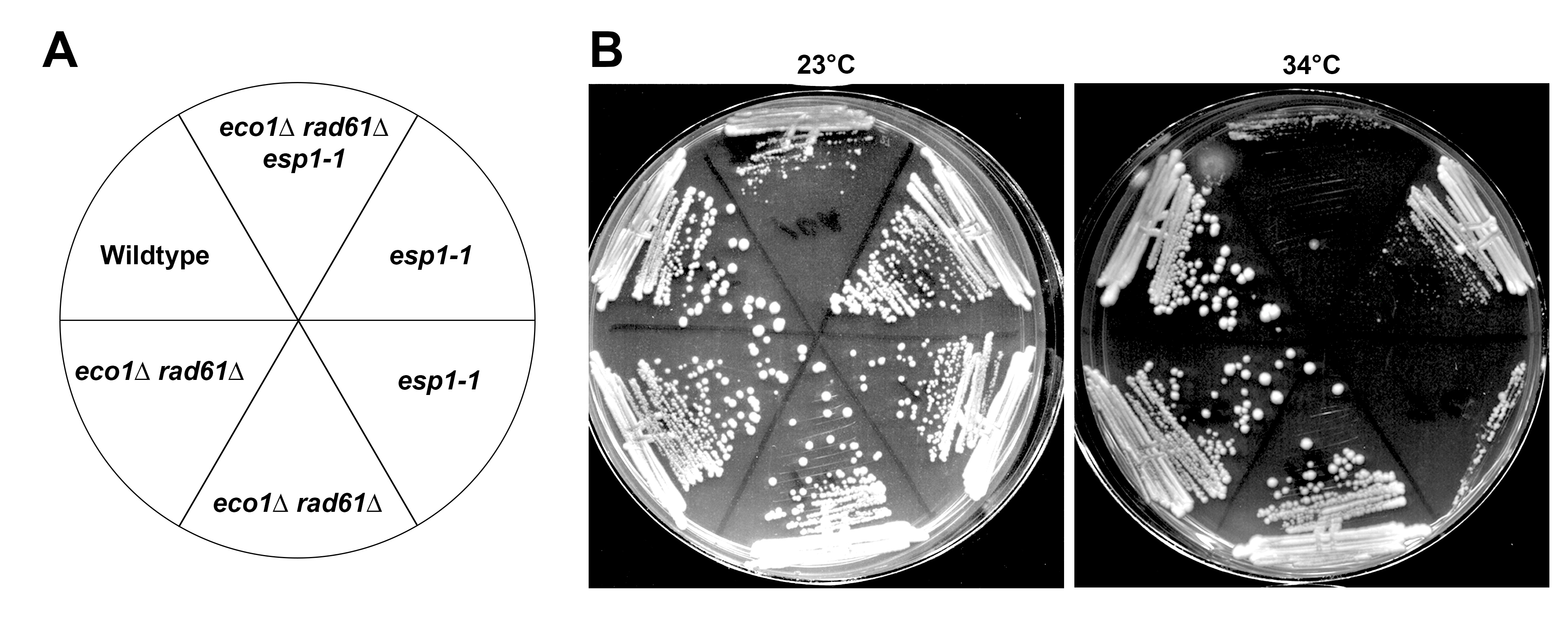

Supplement: S2 Fig — (A) Schematic key for the following strains: wildtype (YPH499), eco1Δ rad61Δ esp1–1 (YBS4864), esp1–1 (YBS4862, YBS4863), eco1Δ rad61Δ (YBS4860, YBS4861). (B) Streak assay for the strains indicated in (A) at 23°C and 34°C. (TIF) [file pgen.1011981.s002.tif]

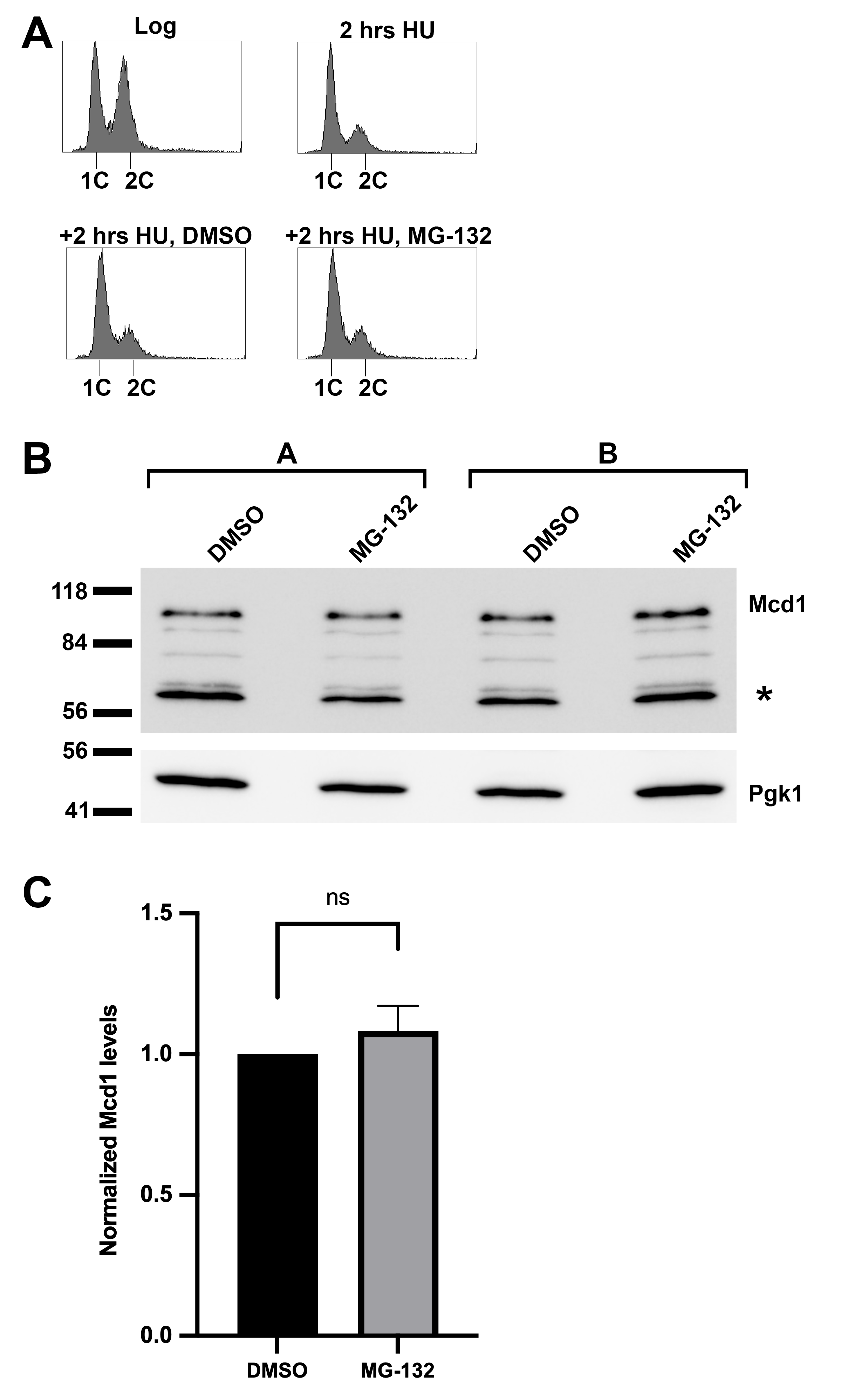

Supplement: S3 Fig — (A) Flow cytometry data of DNA content for log phase of eco1Δ rad61Δ pdr5Δ arrested in S phase at 30°C for 2 hours, then treated with DMSO (control) or MG-132 in the continued presence of HU for an additional 2 hours. (B) Western Blot of Mcd1 (top panel) and Pgk1 (lower panel) protein obtained from extracts of HU-synchronized cells in DMSO or MG132 indicated in (A). “A” and “B” indicate independent biological replicates (YGS375, YGS377). * indicates non-specific band. (C) Quantification of Mcd1, normalized to Pgk1 loading controls. Statistical analysis was performed using a two-tailed t-test. Statistical differences (ns) are based on a P > 0.05 obtained across two biological replicates (n = 2). Error bars indicate the standard deviation. (TIF) [file pgen.1011981.s003.tif]

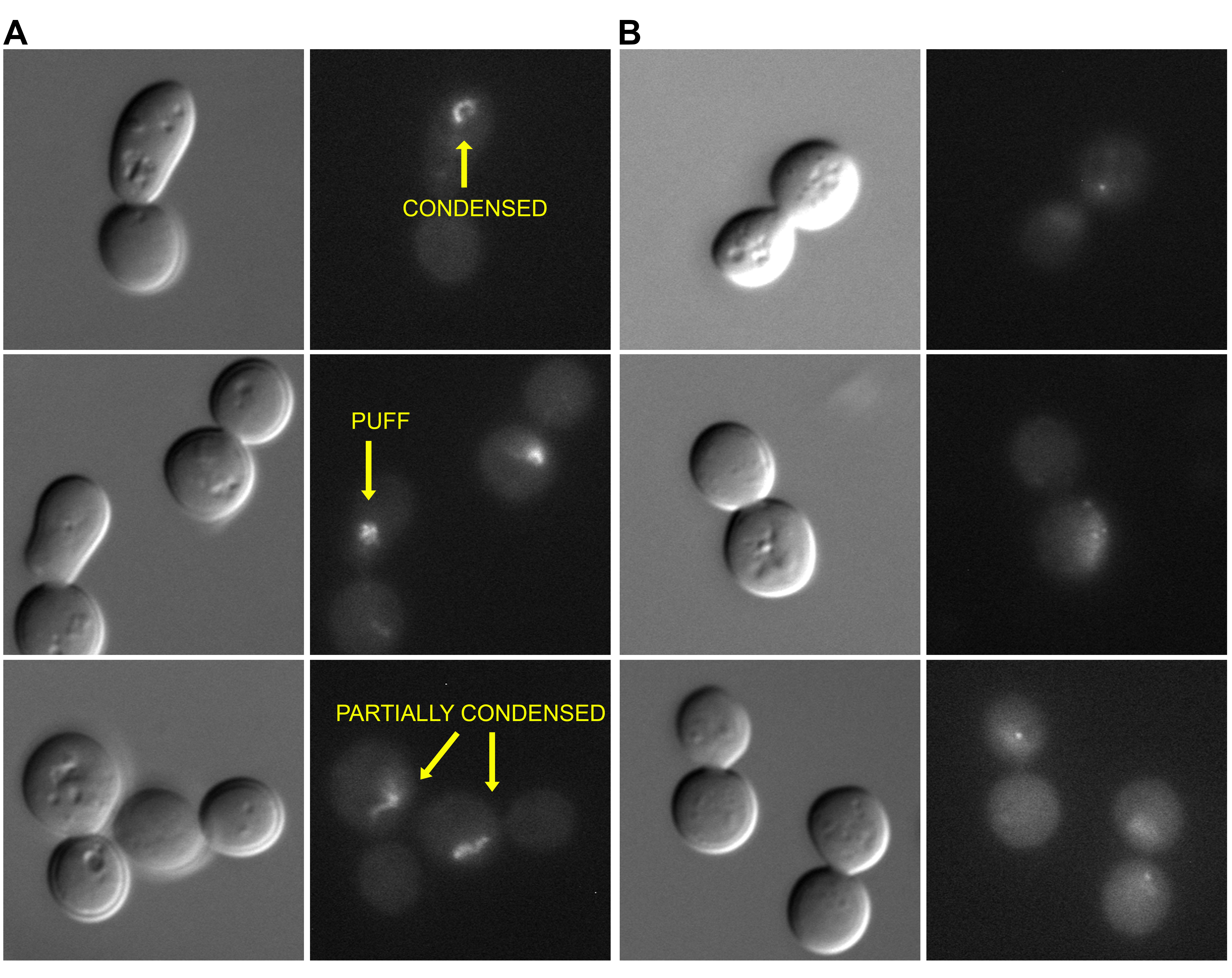

Supplement: S4 Fig — (A) Enlarged micrographs from Fig 8B. Top panel: wildtype cells overexpressing vector alone. Middle panel: smc1–259 cells overexpressing vector alone. Bottom panel: smc1–259 cells overexpressing MCD1. Yellow arrow indicates a condensed, puff, or partially condensed rDNA (see Fig 8 legend). (B) Enlarged micrographs from Fig 9B. Top panel: wildtype cells overexpressing vector alone. Middle panel: smc1–259 cells overexpressing vector alone. Bottom panel: smc1–259 cells overexpressing MCD1 (see Fig 9 legend). (TIF) [file pgen.1011981.s004.tif]
