# Supplementary material for: Aberrant cohesin function in Saccharomyces cerevisiae activates Mcd1 degradation to promote cell lethality
Source: PLoS Genet. 2025 Dec 10;21(12):e1011981. doi: 10.1371/journal.pgen.1011981 (PMC12711053; doi:10.1371/journal.pgen.1011981)
Supplement: S1 Table — (PDF) [file pgen.1011981.s005.pdf]

Strain	Genotype	Reference
YPH499	*MATa ura3-52 lys2-801_amber ade2-101_ochre trp1-Δ63 his3-Δ200 leu2-Δ1*	Sikorski and Hieter, 1989
YBS514	*MATa ura3-52 lys2-801 ade2-101 trp1-Δ63 his3-Δ200 leu2-Δ1 ctf7Δ1::HIS3 ctf7-203:LEU2*	Skibbens, 1999
YMM808	*MATa rad61::URA3 ade2-101 his3Δ200 leu2Δ1 lys2-801 trp1Δ63 ura3-52*	Maradeo and Skibbens, 2010
YMM829	*MATa eco1::HIS3 rad61::URA3 ade2-101 his3Δ200 leu2Δ1 lys2-801 trp1Δ63 ura3-52*	Maradeo and Skibbens, 2010
YMM828	*MATα eco1::HIS3 rad61::URA3 ade2-101 his3Δ200 leu2Δ1 lys2-801 trp1Δ63 ura3-52*	Maradeo and Skibbens, 2010
YGS277	*MATa bul2::TRP1 ura3-52 lys2-801_amber ade2-101_ochre trp1-Δ63 his3-Δ200 leu2-Δ1 (Isolate 1.1)*	This study
YGS279	*MATa eco1::HIS3 rad61::URA3 bul2::TRP1 ade2-101 his3Δ200 leu2Δ1 lys2-801 trp1Δ63 ura3-52 (Isolate2A)*	This study
YGS280	*MATa eco1::HIS3 rad61::URA3 bul2::TRP1 ade2-101 his3Δ200 leu2Δ1 lys2-801 trp1Δ63 ura3-52 (isolate2.1)*	This study
YGS309	*MATa bre1::TRP1 ura3-52 lys2-801_amber ade2-101_ochre trp1-Δ63 his3-Δ200 leu2-Δ1 (isolate b.1)*	This study
YGS292	*MATa eco1::HIS3 rad61::URA3 bre1::TRP1 ade2-101 his3Δ200 leu2Δ1 lys2-801 trp1Δ63 ura3-52 (Isolate 12.1)*	This study
YGS293	*MATa eco1::HIS3 rad61::URA3 bre1::TRP1 ade2-101 his3Δ200 leu2Δ1 lys2-801 trp1Δ63 ura3-52 (Isolate 12.2)*	This study
YGS281	*MATa ldb19::TRP1 ura3-52 lys2-801_amber ade2-101_ochre trp1-Δ63 his3-Δ200 leu2-Δ1 (Isolate 3.1)*	This study
YGS282	*MATa eco1::HIS3 rad61::URA3 ldb19::TRP1 ade2-101 his3Δ200 leu2Δ1 lys2-801 trp1Δ63 ura3-52 (Isolate 4a)*	This study
YGS283	*MATa eco1::HIS3 rad61::URA3 ldb19::TRP1 ade2-101 his3Δ200 leu2Δ1 lys2-801 trp1Δ63 ura3-52 (Isolate 4.2)*	This study
YGS284	*MATa das1::TRP1 ura3-52 lys2-801_amber ade2-101_ochre trp1-Δ63 his3-Δ200 leu2-Δ1 (Isolate 5.1)*	This study
YGS286	*MATa eco1::HIS3 rad61::URA3 das1::TRP1 ade2-101 his3Δ200 leu2Δ1 lys2-801 trp1Δ63 ura3-52 (Isolate 6.1)*	This study
YGS287	*MATa eco1::HIS3 rad61::URA3 das1::TRP1 ade2-101 his3Δ200 leu2Δ1 lys2-801 trp1Δ63 ura3-52 (Isolate 6.3)*	This study

YGS288	*MATa san1::TRP1 ura3-52 lys2-801_amber ade2-101_ochre trp1-Δ63 his3-Δ200 leu2-Δ1 (Isolate 7.1)*	This study
YGS290	*MATa eco1::HIS3 rad61::URA3 san1::TRP1 ade2-101 his3Δ200 leu2Δ1 lys2-801 trp1Δ63 ura3-52 (Isolate 8.2)*	This study
YGS291	*MATa eco1::HIS3 rad61::URA3 san1::TRP1 ade2-101 his3Δ200 leu2Δ1 lys2-801 trp1Δ63 ura3-52 (Isolate 8.3)*	This study
YBS3164/ K699	*MATa ade2-1 trp1-1 can1-100 leu2-3,112 his3-11,15 ura3 GAL psi+*	Gift from Dr. Guacci
YGS209	*MATa ade2-1 trp1-1 can1-100 leu2-3,112 his3-11,15 ura3 GAL psi+ (2µm-TRP1)*	This study
YGS210	*MATa ade2-1 trp1-1 can1-100 leu2-3,112 his3-11,15 ura3 GAL psi+ (2µm TRP1 MCD1)*	This study
YBS3170/ K5824	*MATa smc3-42 ade2-1 trp1-1 can1-100 leu2-3,112 his3-11,15 ura3 GAL psi+*	Gift from Dr. Guacci
YGS229	*MATa smc3-42 ade2-1 trp1-1 can1-100 leu2-3,112 his3-11,15 ura3 GAL psi+ (2µm-TRP1)*	This study
YGS230	*MATa smc3-42 ade2-1 trp1-1 can1-100 leu2-3,112 his3-11,15 ura3 GAL psi+ (2µm TRP1 MCD1) (Isolate 6A)*	This study
YGS231	*MATa smc3-42 ade2-1 trp1-1 can1-100 leu2-3,112 his3-11,15 ura3 GAL psi+ (2µm TRP MCD1) (Isolate 6B)*	This study
YBS3168/ K6013	*MATa smc1-259 ade2-1 trp1-1 can1-100 leu2-3,112 his3-11,15 ura3 GAL psi+*	Gift from Dr. Guacci
YGS211	*MATa smc1-259 ade2-1 trp1-1 can1-100 leu2-3,112 his3-11,15 ura3 GAL psi+ (2µm-TRP)*	This study
YGS212	*MATa smc1-259 ade2-1 trp1-1 can1-100 leu2-3,112 his3-11,15 ura3 GAL psi+ (2µm TRP1 MCD1) (Isolate 1)*	This study
YGS213	*MATa smc1-259 ade2-1 trp1-1 can1-100 leu2-3,112 his3-11,15 ura3 GAL psi+ (2µm TRP1 MCD1) (Isolate 2)*	This study
YGS329	*MATa ura3-52 lys2-801 ade2-101 trp1-Δ63 his3-Δ200 leu2-Δ1 ctf7Δ1::HIS3 ctf7-203:LEU2 + (2µm-TRP1)*	This study
YGS330	*MATa ura3-52 lys2-801 ade2-101 trp1-Δ63 his3-Δ200 leu2-Δ1 ctf7Δ1::HIS3 ctf7-203:LEU2 + (2µm TRP1 MCD1) (Isolate A)*	This study
YGS331	*MATa ura3-52 lys2-801 ade2-101 trp1-Δ63 his3-Δ200 leu2-Δ1 ctf7Δ1::HIS3 ctf7-203:LEU2 + (2µm TRP1 MCD1) (Isolate B)*	This study
YGS335	*MATa NET1-GFP:kanMX6 ade2-1 trp1-1 can1-100 leu2-3,112 his3-11,15 ura3 GAL psi+ (2µm-TRP1)*	This study

YGS337	*MATa NET1-GFP:kanMX6 ade2-1 trp1-1 can1-100 leu2-3,112 his3-11,15 ura3 GAL psi+ (2µm TRP1 MCD1)*	This study
YGS338	*MATa smc1-259 NET1-GFP:kanMX6 ade2-1 trp1-1 can1-100 leu2-3,112 his3-11,15 ura3 GAL psi+ (2µm-TRP1)*	This study
YGS341	*MATa smc1-259 NET1-GFP:kanMX6 ade2-1 trp1-1 can1-100 leu2-3,112 his3-11,15 ura3 GAL psi+ (2µm TRP1 MCD1)*	This study
YBS1042	*MATα ade2 trp1 his3 leu2::LEU2tetR-GFP ura3::3xURA3tetO112 PDS1-13MYC:TRP1*	Kenna and Skibbens, 2003
YGS333	*MATa ade2 trp1 leu2::LEU2tetR-GFP ura3::3xURAtetO112 trp1 + (2µm-TRP1) (spore A4B)*	This study
YGS334	*MATa ade2 trp1 leu2::LEU2tetR-GFP ura3::3xURAtetO112 trp1 + (2µm TRP1 MCD1) (spore A4B)*	This study
YGS321	*MATa smc1-259 ade2 trp1 leu2::LEU2tetR-GFP ura3::3xURAtetO112 trp1 + (2µm-TRP1) (sporeE1B)*	This study
YGS323	*MATa smc1-259 ade2 trp1 leu2::LEU2tetR-GFP ura3::3xURAtetO112 trp1 + (2µm TRP1 MCD1) (sporeE1B)*	This study
3327-9B	*Mata scc3-6 GFPLacI-HIS3 his3-11,15 trp1-1 leu2-3,112 ura3-52 bar1 GAL+*	Gift from Dr. Guacci
YBS4558	*MATa ura3-52 lys2-801_amber ade2-101_ochre trp1-Δ63 his3-Δ200 leu2-Δ1 + (2µm URA3)*	This study
YBS4562	*MATa ura3-52 lys2-801_amber ade2-101_ochre trp1-Δ63 his3-Δ200 leu2-Δ1 + (2µm URA3 MCD1)*	This study
YBS4567	*Mata scc3-6 GFPLacI-HIS3 his3-11,15 trp1-1 leu2-3,112 ura3-52 bar1 GAL+ + (2µm URA3)*	This study
YBS4568	*Mata scc3-6 GFPLacI-HIS3 his3-11,15 trp1-1 leu2-3,112 ura3-52 bar1 GAL+ + (2µm URA3 MCD1)*	This study
YBS4569	*Mata scc3-6 GFPLacI-HIS3 his3-11,15 trp1-1 leu2-3,112 ura3-52 bar1 GAL+ + (2µm URA3 MCD1)*	This study
KT048	*MATa scc2-4 MCD1-3HA:TRP1 ade2-1 his3-11,15 leu2-3,112 trp1-1 ura3-1 can1-100*	Tong and Skibbens, 2014
KT046	*MATa MCD1-3HA:TRP1 ade2-1 his3-11,15 leu2-3,112 trp1-1 ura3-1 can1-100*	Tong and Skibbens, 2014
YGS216	*MATa MCD1-3HA:TRP1 ade2-1 his3-11,15 leu2-3,112 trp1-1 ura3-1 can1-100 + (2µm LEU2)*	This study
YGS217	*MATa MCD1-3HA:TRP1 ade2-1 his3-11,15 leu2-3,112 trp1-1 ura3-1 can1-100 + (2µm LEU2 MCD1)*	This study
YGS218	*MATa scc2-4 MCD1-3HA:TRP1 ade2-1 his3-11,15 leu2-3,112 trp1-1 ura3-1 can1-100 + (2µm LEU2)*	This study

YGS219	*MATa scc2-4 MCD1-3HA:TRP1 ade2-1 his3-11,15 leu2-3,112 trp1-1 ura3-1 can1-100 + (2μm LEU2 MCD1) (Isolate 1)*	This study
YGS220	*MATa scc2-4 MCD1-3HA:TRP1 ade2-1 his3-11,15 leu2-3,112 trp1-1 ura3-1 can1-100 + (2μm LEU2 MCD1) (Isolate 2)*	This study
BB2788	*MATa esp1-1 ura3 leu2 can1-100 lys2*	Gift from Dr. Guacci
YGS344	*MATa esp1-1:kanMX6 ura3-52 lys2-801_amber ade2-101_ochre trp1-Δ63 his3-Δ200 leu2-Δ1*	This study
YGS348	*MATa esp1-1:kanMX6 ura3-52 lys2-801_amber ade2-101_ochre trp1-Δ63 his3-Δ200 leu2-Δ1*	This study
**YBS255**	*MATa CTF7:LEU2 ctf7::HIS3 ade2-101 his3Δ200 leu2Δ1 lys2-801 trp1Δ63 ura3-53*	Skibbens, 1999
**YMM828**	*MATalpha cft7::HIS3 rad61::URA3 ade2-101 his3Δ200 leu2Δ1 lys2-801 trp1Δ63 ura3-52*	Maradeo and Skibbens, 2010
**YBS4067**	*MATa CTF7:LEU2 ctf7::HIS3 ade2-101 his3Δ200 leu2Δ1 lys2-801 trp1Δ63 ura3-53 + (2μm TRP1)*	This study
**YBS4071**	*MATa CTF7:LEU2 ctf7::HIS3 ade2-101 his3Δ200 leu2Δ1 lys2-801 trp1Δ63 ura3-53 + pGS2 (2μm TRP1 PDS5)*	This study
**YBS4069**	*MATalpha cft7::HIS3 rad61::URA3 ade2-101 his3Δ200 leu2Δ1 lys2-801 trp1Δ63 ura3-52 + (2μm TRP1)*	This study
**YBS4075**	*MATalpha cft7::HIS3 rad61::URA3 ade2-101 his3Δ200 leu2Δ1 lys2-801 trp1Δ63 ura3-52 + pGS2 (2μm TRP1 PDS5) iso A*	This study
**YBS4076**	*MATalpha cft7::HIS3 rad61::URA3 ade2-101 his3Δ200 leu2Δ1 lys2-801 trp1Δ63 ura3-52 + pGS2 (2μm TRP PDS5) iso B*	This study
**YBS2031/ KT046**	*MATa ade2-1 his3-11,15 leu2-3,112 trp1-1 ura3-1 can1-100 MCD1-3HA:TRP1*	Tong and Skibbens, 2014
**YBS2033/ KT048**	*MATa ade2-1 his3-11,15 leu2-3,112 trp1-1 ura3-1 can1-100 scc2-4 MCD1-3HA:TRP1*	Tong and Skibbens, 2014
**YBS4864**	*MATα eco1::HIS3 rad61::URA3 esp1-1:kanMX6 ade2-101 his3Δ200 leu2Δ1 lys2-801 trp1Δ63 ura3-52 (spore 10A)*	This study
**YBS4862**	*MATa esp1-1:kanMX6 ura3-52 lys2-801_amber ade2-101_ochre trp1-Δ63 his3-Δ200 leu2-Δ1 (spore 7C)*	This study
**YBS4863**	*MAT esp1-1:kanMX6 ura3-52 lys2-801_amber ade2-101_ochre trp1-Δ63 his3-Δ200 leu2-Δ1 (spore 3B)*	This study
**YBS4860**	*MATa eco1::HIS3 rad61::URA3 ade2-101 his3Δ200 leu2Δ1 lys2-801 trp1Δ63 ura3-52 (spore 4C)*	This study
**YBS4861**	*MATα eco1::HIS3 rad61::URA3 ade2-101 his3Δ200 leu2Δ1 lys2-801 trp1Δ63 ura3-52 (spore 9D)*	This study

**YGS375**	*MATalpha eco1::HIS3 rad61::URA3 pdr5::TRP ade2-101 his3∆200 leu2∆1 lys2-801 trp1∆63 ura3-52*	This study
**YGS377**	*MATa eco1::HIS3 rad61::URA3 pds5::TRP ade2-101 his3∆200 leu2∆1 lys2-801 trp1∆63 ura3-52*	This study
**YGS352**	*MATa ubr1::kanMX6 ura3-52 lys2-801_amber ade2-101_ochre trp1-Δ63 his3-Δ200 leu2-Δ1 (Isolate c1)*	This study
**YGS354**	*MATalpha ubr1::kanMX6 eco1::HIS3 rad61::URA3 ade2-101 his3∆200 leu2∆1 lys2-801 trp1∆63 ura3-52 (Isolate e1)*	This study
**YGS355**	*MATalpha ubr1::kanMX6 eco1::HIS3 rad61::URA3 ade2-101 his3∆200 leu2∆1 lys2-801 trp1∆63 ura3-52 (Isolate e2)*	This study
