# Supplementary material for: Aberrant cohesin function in Saccharomyces cerevisiae activates Mcd1 degradation to promote cell lethality
Source: PLoS Genet. 2025 Dec 10;21(12):e1011981. doi: 10.1371/journal.pgen.1011981 (PMC12711053; doi:10.1371/journal.pgen.1011981)
Supplement: S2 Table — (PDF) [file pgen.1011981.s006.pdf]

Name (oRVS)	DNA oligos Sequence 5' to 3'
8	ACG ACT CAT CTC CAT GCA GTT
23	GAT TGT CGC ACC TGA TTG CC
3370	AGG ACT TTG CAA CTG AAG CAG CAG ATT TGA GAT ATA TTC TGG GGA ACA AAA GAA GTA TTA CGG ATC CCC GGG TTA ATT AA
3371	ATA GTC CTG CTT TTA TCA ATT ATT TGT AAA ACT GCG AGA TTA CTG TTA GTG TTG TAT GGT GAATTC GAG CTC GTT TAA AC
3372	GTC CAA CAG ATA TGC GTA AAG
3373	CAG AAA TTG CTG ATT TTT ACT CCT ACT TAG TAT ACA TTT CAC TAA ACA ATA CGT TTT ACC CGGATC CCC GGG TTA ATT AA
3374	AGT TTT CAG GTA TCT AAG ATA AAA ATA TAT GGT AAA TAC CTT TAA CGA ATA TTA TAA AAT GAATTC GAG CTC GTT TAA AC
3375	TTG CTT ACT TGT ATC GCT ATT
3376	CCG TGC AAA ATA TCC AGG ACG TCT ATA CAC AGT GTT TAC AAC TCA GCT TAT ATT CAT ATC CGG ATC CCC GGGTTA ATT AA
3377	CTA GCT TAA AAA ATG CGT TGA ATA TAT ATT ATT AAA TAT ATA TAT TTG AAG GGG AGT TGA GAA TTC GAG CTCGTT TAA AC
3378	TGT TCA CCA GAG GAT TCT TTC
3379	TTC TCC CTT TTT TCC CCT TTG TTT TCT CTC ATA GTC TTG TAA CCT CAG CTT TTG TTC ATT CGG ATC CCC GGG TTA ATT AA
3380	AAA TGG ATG ACT GCC AAT AGG ACA TAT TTT CAT ATT AAC ATA CTT CAG AAG CGG TAT TGT GAA TTC GAG CTC GTT TAA AC
3381	ATA CTT CGA CGC AAA AAG CCG
3385	AGA GAT AGA AAG GGC TTT CAC CGT TTT TAT GCT AAT CGT GCT AGC TGA TAA TAA TCA GAT CGG ATC CCC GGG TTA ATT AA
3386	TAT GTA TAT GTA TGT GGA GGA TAT AAC ACA AAC AGT GGA AAA GTG GTA GAA TAA TTA GTA GAA TTC GAG CTC GTT TAA AC
3387	CTG GTG AAA TTC TGA GAT CGT
1203	TTT AGG TAA GAA GAA GAA GCC AAG TGG TGG ATT TGC ATC ATT AAT AAA AGA TTT CAA GAA AAA ACG GAT CCC CGG GTT AAT TAA
1204	TGC TTG ATT ATT TTT TTT TAC TAG CTT TCT GTG ACG TGT ATT CTA CTG AGA CTT TCT GGT ATC AGA ATT CGA GCT CGT TTA AAC
3390	CTT GTT TCA AGA GGC ATC CCA
3346	GGGAAGAAGGAACAAGACAAA (qPCR *MCD1* sense)
3347	TCCCTAGATCCCAACCAATAG (qPCR *MCD1* antisense)
3348	CAGGCAATGTCACGGATAG (qPCR *ALG9* sense)
3349	CCTTCACACCACCTTGATTTA (qPCR *ALG9* antisense)
