# Supplementary material for: Aberrant cohesin function in Saccharomyces cerevisiae activates Mcd1 degradation to promote cell lethality
Source: PLoS Genet. 2025 Dec 10;21(12):e1011981. doi: 10.1371/journal.pgen.1011981 (PMC12711053; doi:10.1371/journal.pgen.1011981)
Supplement: Attachment — Submitted filename: Response_Singh.pdf [file pgen.1011981.s008.pdf]

Dear Profs van Werven, Butler, Dudley and Goriely,

We thank each of you and our reviewers for their time and effort on our behalf. We trust that you will find that we have been diligent in responding to all reviewer comments and suggestions. Included in the revised study are several new experiments that cover the generation of new strains, Western blots, use of proteosome inhibitors, etc. We are pleased that each support the models stated in our original submission.

We are excited by this study in describing evidence for a checkpoint that directly targets the cohesin subunit, Mcd1/Scc1. Given the clinical relevance of checkpoints in cancer therapies, this study represents the most significance advance since the discovery of cohesin extrusion functions - roughly 8 years ago. We also note that our findings will significantly impact all prior analyses of cohesin mutated genes - given that phenotypes originally ascribed to those particular alleles are in large part due to reduced levels of wildtype Mcd1/Scc1. Finally, the discovery of a feedback mechanism through which cells regulate Mcd1 protein levels is likely to launch new studies of cohesin biology.

Below we reproduce in full reviewers' comments, followed immediately by our responses (in *italics*). We thank you again for your time and efforts in shepherding this study to completion.

Cheers,

Bob and Gurvir

Reviewer #1

In this study Singh et al propose that mcd1 protein is downregulated by E3 ligase mediated targeting in cohesin mutant yeast cells. To demonstrate this, they delete various E3 ligases in eco1Δ rad61Δ and show that deletion of two ligases reduces the growth defect observed in eco1Δ rad61Δ. In cells that contain mutation for other cohesin genes (eco1-1, scc3-6, smc3-42, and smc1-259) they overexpress mcd1to rescue the cohesin mutation associated defects.

The data regarding E3 ligase mediated targeting is not convincing.

Major points

1. Were the protein/RNA levels of other cohesin subunit/genes examined in eco1Δ rad61Δ? It will be good to show the levels of other cohesin subunits to determine whether only mcd1 is altered.

The reviewer raises an excellent point - ensuring that other cohesin proteins remain unaltered in eco1Δ rad61Δ is an important tenet of our model. To address this, wildtype and eco1Δ rad61Δ cells were arrested in S phase (see Fig A, below). Following cell lysis, Mcd1, Pds5 and Pgk1 (loading control) protein levels were assessed by

Western blotting. As expected, Mcd1 levels were reduced in the eco1 rad61 cells compared to wildtype (see Fig B, below). In contrast, quantification of Pds5 protein levels normalized to Pgk1 revealed no significant differences in Pds5 protein levels in wildtype versus eco1 rad61 cells (see Figs B and C below). Statistical significance was assessed using a two-tailed t test, n.s, P > 0.05.

We are aware that Pds5 levels might be reduced at levels not easily detectable as significant by Western blot. If Pds5 protein levels are indeed reduced and thus responsible for phenotypes exhibited by eco1Δ rad61Δ cells, then we would expect that elevated levels of Pds5 would suppress eco1Δ rad61Δ cell ts growth defects similar to the suppression achieved upon Mcd1 elevated expression (Singh and Skibbens 2024). To test this possibility, wildtype and eco1Δ rad61Δ cells were transformed with vector alone or vector driving elevated expression of PDS5. Log phase culture of the resulting transformants were then serially diluted onto selective media plates. Overexpression of PDS5 appeared to have no negative impact on wildtype cell growth. Importantly, elevated expression of PDS5 failed to restore the temperature-sensitive growth defects exhibited by eco1Δ rad61Δ cells (see Fig D, below).

Together, these results suggest that Mcd1 is uniquely targeted for degradation and that the observed rescue that occurs due to MCD1 overexpression is specific to

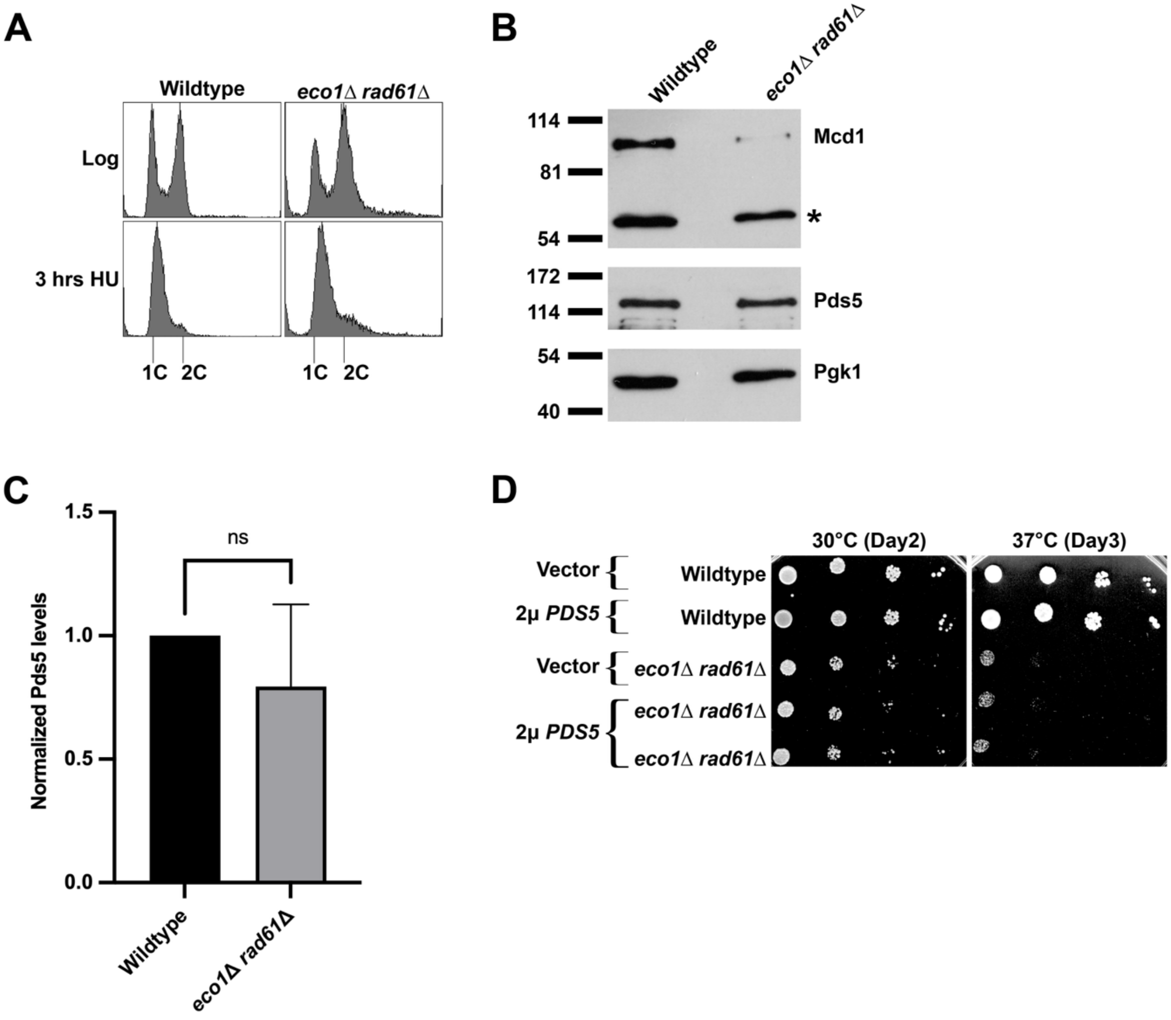

restoring Mcd1 levels. These findings are incorporated into the revised manuscript and added as a new supplemental figure (Fig S1).

2. To show mcd1 targeting by E3 ligases, mcd1 protein/RNA need to be examined in in eco1Δ rad61Δ and other cohesin mutant cells in which ligases have been deleted.

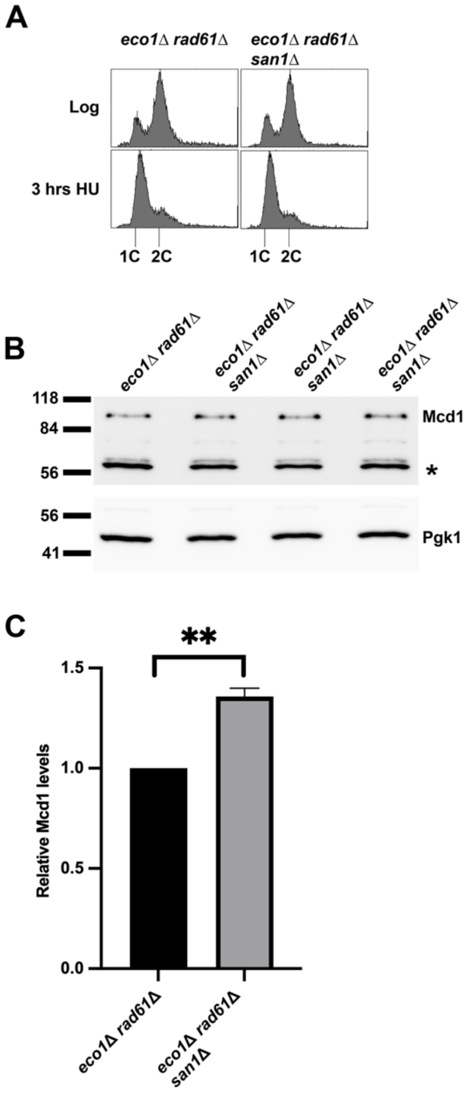

*We are sure that the reviewers and editors appreciate the challenges involved in identifying degradation pathways through which Mcd1 is targeted. Our genetic analyses regarding SAN1 represents an important step forward and, we argue, if fairly compelling. We agree, however, that testing whether deletion of SAN1 elevates Mcd1 protein levels in eco1Δ rad61Δ cells represents a second and critical test of our model. To address this comment, eco1Δ rad61Δ cells and 3 biological replicates of the eco1Δ rad61Δ san1Δ triple mutant cells were arrested in S phase (see accompanying Fig A). Following cell lysis, Mcd1 and Pgk1 (loading control) protein levels were assessed by Western blot. Quantification of Mcd1 protein levels normalized to Pgk1 revealed that SAN1 deletion elevated Mcd1 protein levels by ≈35% in eco1Δ rad61Δ mutant cells. (see accompanying Figs B and C). Statistical significance was assessed using a one sample two tailed t-test (**, P ≤ 0.01). These findings support the model that San1 is responsible for reducing Mcd1 proteins levels. These results are now included in the revised M.S as a new Figure 5.*

3. It is unclear as to whether the authors are suggesting that E3 ligase activity is altered in cohesin defective state.

We apologize for the lack of clarity and thank the reviewer for providing us with the opportunity to make our findings more clear. In summarizing our results, we provide evidence that Mcd1 is degraded in response to defects in cohesin function - even in cells that retain wildtype versions of every core cohesin subunit gene. We further provide evidence that degradation of cohesin occurs through a mechanism previously not associated with Mcd1 turnover and that occurs independent of the APC/Esp1 pathway (the mechanism through which Mcd1 is cleaved at anaphase onset). Given that Mcd1 levels are reduced, despite a significant (over 5-fold) increase MCD1 transcription, indicates that the degradation pathway described here must be elevated to a significant level. How signals relayed to E3 ligases, in response to cohesin deficiencies, will constitute the

next important advancement in cohesin biology. In the revised ms, we discuss several possibilities. For instance, E3 ligases might become active in response to the retention of an active spindle assembly checkpoint after mitosis. Another possibility is that cohesin defects result in transcriptional dysregulaton that directly impact Mcd1 expression. In either case, there is strong evidence that CDK plays a critical role. For instance, deletion of a G1 cyclin (CLN2) rescues both eco1Δ rad61Δ cell ts growth defects and pds5Δ elg1Δ lethality - both by increasing Mcd1 levels (Buskirk and Skibbens, 2023; Choudhary et al., 2023, Singh and Skibbens, 2024). Discerning how cohesin defects activate E3 ligases such as San1, potentially via G1-CDKS, represents an exciting new intersection between cohesin biology and cell cycle progression to explore.

4. For the mcd1 overexpression experiments, it will be good to know what happens in eco1Δ rad61Δ cells. It will also be good to show mcd1 levels before and after overexpression of mcd1 in the wild type and cohesin mutant cells tested.

We agree that demonstrating a rescue of eco1Δ rad61Δ cells (that have no compromised structure) through MCD1 overexpression strengthens our model that cohesin dysfunction, rather than altered cohesin structure, drives both Mcd1 degradation and cell lethality. Notably, our previous work directly assessed this point, overexpression of MCD1 fully restores the viability (even to 37°C) of eco1Δ rad61Δ cells (Singh and Skibbens, 2024)

With regards to the reviewer's second question about Mcd1 levels, we point to multiple studies that describe the 2 μm plasmid as a high copy vehicle that effectively elevates protein levels (originally described in Futcher 1986, J. Theor Biol; Chan et al., 2013, but present in hundreds of studies through which protein levels are increased). We anticipate that Mcd1 levels are highly increased and maintained in both wildtype and eco1Δ rad61Δ cells, a supposition borne out by the evidence that 2 μm plasmids that harbor MCD1 indeed greatly suppress the temperature sensitive growth of every cohesin mutated strain tested (our current study and Buskirk and Skibbens, 2023; Choudhary et al., 2023; Singh and Skibbens, 2024). Conversely, it is not clear to us the extent to which quantifying Mcd1 levels in each of these strains will provide useful information. For this first report of these suppressions - the finding itself will be of immense value to the research community.

5. Mcd1 downregulation cannot be the primary mechanism for the defects seen in cells with cohesin gene mutation. It is unclear whether the authors are suggesting that mcd1 protein regulation is the primary mechanism or adds to the defects. This needs to be clarified in the manuscript. In cells with defects in core cohesin subunit like smc1-259 , the authors need to clarify how mcd1 overexpression partially restores cohesion. Even if mcd1 is increased there is still reduced smc1 and hence reduced components to form the core ring complex.

This reviewer offers a complicated comment - thus we want to respond to the issues raised in the following ways. First, we want to clarify the state of the literature. There is no evidence to date that Smc1 protein levels are indeed reduced in smc1-259 mutant cells (Kimm, 2002; Michaelis, 1997; Frattini, 2017). Regardless, the evidence is clear that deletion of SMC1 is lethal and that mutation of SMC1 appears to result in several phenotypes: temperature sensitivity and sister chromatid cohesion defects (Frattini, 2017), as well as condensation defects (newly reported in our current study).

Second - we apologize for not making the model more clear. In response, we revised the manuscript to clarify that, based on the findings from this study and previous reports, Mcd1 levels are reduced in various cohesin mutant cells (smc1-259, smc3-42, eco1-1, pds5-1, scc2-4, eco1 rad61) and that this reduction plays a physiologically relevant impact on those cells. Subsequently, the critical question then was to determine the extent to which reduced Mcd1 levels contribute to the the various phenotypes associated with cohesin mutations - focusing on an allele of SMC1. In the revised manuscript, we make clear that the suppression of smc1-259 temperature cell growth defects results from the restoration of Mcd1 protein levels. Third, we pursue these findings by demonstrating that the degradation of Mcd1 in smc1-259 cells exacerbates primarily the cohesion defects otherwise exhibited by these cells, with less of an impact on condensation defects. We conclude that restoring Mcd1 levels rescues cell viability by partially recovering at least two cohesin dependent processes- cohesion (primarily) and condensation (to a lesser extent).

6. The impact of reduced Esp1 has been stated as data not shown. It will be good to show this data either in the main fig or supplementary data.

We apologize for not including this information in the original submission. That data is now included as Supplemental Figure 2.

Minor point

Figure 5B and 6B it would be better to have more zoomed in images. In figure 5B examples of partial and puff should be provided.

We added the zoomed-in micrographs (Supplementary Figure 4) that better highlight the effects of MCD1 overexpression on sister chromatid cohesion and rDNA condensation in smc1-259 cells. The rDNA puffs and partially condensed rDNA are labelled using a yellow arrow.

<u>Reviewer #2</u>

Mcd1 is an essential subunit of cohesin that is regulated throughout the cell cycle. Several studies, including previous work from this lab, have shown that Mcd1 is degraded when cohesin complex integrity is compromised. In this paper, Singh and Skibbens explore Mcd1 stability in eco1Δ wpl1Δ cells, in which chromatin-bound cohesin levels are increased, and find that under these conditions, Mcd1 levels still

decrease. This finding led the authors to suggest that an active mechanism regulates Mcd1 levels. They identified ubiquitin-related factors that mediate Mcd1 degradation. In addition, they showed that reduced Mcd1 levels in Smc1 ts mutant impair cohesion but not chromosome condensation.
Cohesin is a key factor in chromatin organization, and this work suggests an interesting new paradigm for its regulation. Strengthening the mechanistic evidence would enhance the study's impact.

Major Issues

1. The regulation of Mcd1 by San1 and Das1 is shown genetically. The identification of physical interaction between these factors is based on proteomics, as described in Litwin et al., NAR, 2023. However, the confidence in the results is low, and these specific interactions have not been validated. Molecular evidence is required to support the interplay between these factors and cohesin.

We appreciate the reviewer's concern, but point out that Litwin, 2023 is not the only study to identify a physical interaction between the San1 E3 ligase and cohesins through affinity capture-mass spectroscopy. An independent study also reported a physical interaction between cohesin and San1 (Yang X, 2022). These prior studies, published in PNAS and Nucleic Acids Research, are at least consistent with our findings that SAN1 deletion improves cell viability of the eco1Δ rad61Δ cells. However, the reviewer's point is well taken - thus we decided to explore further this connection.

We addressed the reviewer's concerns in two ways. First, we examined Mcd1 levels by Western blot in eco1Δ rad61Δ cells and eco1Δ rad61Δ san1Δ cells. Our new results reveal that SAN1 deletion indeed increases Mcd1 protein levels otherwise exhibited by eco1Δ rad61Δ cells, providing strong support that San1 targets Mcd1 for degradation. Second, we tested whether the proteosome (the downstream 'executive' player in ubiquitin-targeted degradation) played a critical role in Mcd1 degradation. As described in greater detail below, eco1Δ rad61Δ cells incubated in the presence of the proteosome inhibitor, MG132, exhibited a significant increase in Mcd1 levels, compared to vehicle alone. In combination, these new experiments support the interplay between Mcd1 degradation, San1 E3 ligase, and the proteosome.

2. The results show that Mcd1 overexpression in the smc1-259 background restores the cohesion defect. To better understand the mechanism, a ChIP assay should be performed to evaluate the impact on the chromatin-bound cohesin levels.

We are sure that the reviewer knows that smc1-259 cells exhibit both cohesin defects as well as reduced Mcd1 binding to chromatin (Michaelis 1997; Frattini, 2017). It is difficult to consider a model in which the reviewer might suggest that elevating Mcd1 levels in smc1-259 cells rescues their ts growth defects without restoring *cohesin recruitment to chromatin. Moreover, these are not trivial experiments. Prior chip experiments on cohesins comprise the major findings in multi-authored Cell and*

Molecular Cell papers - well beyond (and separate from) the intent of this current study and the available manpower (and funds).

We further submit that restoration of cohesin-DNA binding is expected, it cannot fully account for the observed rescue, as previous studies have shown that hyper-stabilizing cohesins on DNA by ECO1 overexpression does not improve smc1-259, smc3-42, pds5-3, mcd1-73 cell viability (Jingjing Zhang, 2017). Nor does increasing cohesin stability onto DNA, via RAD61 deletion, suppress the temperature sensitivity of smc3-42 or mcd1-1 cells (Thomas Eng, 2015). Thus, the ChIP experiment proposed by the reviewer is unlikely to provide mechanistic insights. We ask that the publishing that the current study should not be delayed due to the intensive ChIP experiments suggested by the reviewer.

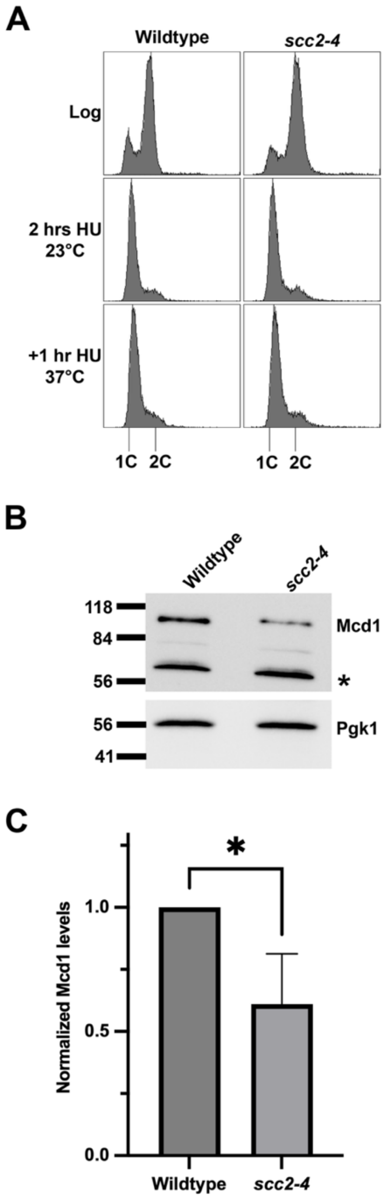

3. The first part of the study uses eco1Δ wpl1Δ cells, whereas the cell biology experiments were performed in smc1-259 cells. While Eco1 Wpl61 are regulators that are not part of the cohesin core, Smc1 is a core subunit. It hasn't been established that cohesin complex integrity is uncompromised in smc1-259 cells and thus, that the phenotypes are fully dependent on Mcd1 degradation and not in the stability of the holocomplex. This raises the question of whether these strains share the same mechanism. The integrity of the cohesin with the smc1-259 allele in restrictive conditions should be tested.

Respectfully, we do not claim that the phenotypes exhibited by smc1-259 cells are fully dependent on Mcd1 degradation. This is patently not true since smc1-259 cells are inviable at 37°C even in the presence of elevated expression of MCD1 (ie - MCD1 expression significantly suppresses the ts growth defect - but does bypass Smc1 function). What is true is that elevated levels of MCD1 significantly suppress the growth defects of both smc1-259 cells as well as eco1Δ rad61Δ cells.

We agree, however, that we cannot fully rule out the possibility that some level of structural integrity is compromised. Even in this scenario, however, that smc1-259 cells target Mcd1 for degradation supports our model that cells monitor for reduced cohesin function.

To further address the reviewer's comment, we decided to test the extent to which cells monitor for "functional defects", and in turn, activate the degradation machinery that targets Mcd1. Here, we examined Mcd1 protein levels in scc2-4 mutant

cells. Scc2 is critical for cohesin loading, but it is not a core nor auxiliary component of the cohesin complex (Ciosk et al., 2000; Lopez-Serra et al., 2015). More importantly, plays no role in cohesin assembly. To test whether Mcd1 protein levels are altered in cohesin deposition-deficient cells that harbor wildtype versions of every core cohesin subunit (and also ECO1 and RAD61), wildtype and scc2-4 cells were arrested in S phase for 2 hours at 23°C (permissive temperature) and then shifted to 37°C for 1 hour to inactivate scc2 (FACS profiles shown in the accompanying Fig A). Following cell lysis, Mcd1 and Pgk1 (loading control) protein levels were analyzed by Western blot. Quantification of Mcd1 normalized to Pgk1 revealed that scc2-4 inactivation led to a significant reduction in Mcd1 protein levels compared to wild-type cells (see Figs B and C). These findings indicate that even if the cohesin complex integrity is preserved, functionally impairing cohesin activity is sufficient to promote Mcd1 degradation. These findings augment our findings that cohesin-defective cells (eco1-1, eco1Δ rad61Δ, and rad61Δ), that all harbor wildtype version of cohesin genes, also promote Mcd1 degradation. We ask that publication of our current findings not be hampered by a requirement to reproduce the arduous and tour-de-force biochemical cohesin analyses reported by the Nasmyth lab in Cell/Molecular Cell. [you can certainly delete this last sentence if you wish ...]

4. Guacci et al. reported condensation defects in cells carrying the smc3-RR allele. Does Mcd1 overexpression suppress this allele as well?

As the reviewer knows, the smc3-RR mutation bypasses a requirement for Eco1-dependent cohesion establishment, but exhibits chromosome condensation defects. While MCD1 elevated expression suppresses the condensation defect exhibited by smc1-259 cells (first reported in our current study) and also eco1Δ rad61Δ ts growth defects, the experiment suggested by the reviewer appears to be more about differentiating between how various cohesin gene mutations impact condensation. This is not the point of our study and we ask that determining the extent to which Mcd1 loss contributes to cohesion establishment or chromosome condensation in smc3-RR or across the span cohesin mutants (which will require a prolonged systematic approach) is well beyond the scope of the current study.

Minor Points

1. Line 148: “data not shown” - all data, including negative results, should be included. Provide these results in the supplementary material.

We apologize for the error regarding journal requirements. All data are now included either in the main body of the study or as supplemental information.

2. Lines 216–217: “Surprisingly, MCD1 expression produced only a modest increase” - state whether this modest increase is statistically significant.

We thank the reviewer for the opportunity to clarify the results. The text has been revised to state that "Surprisingly, MCD1 expression produced only a modest (albeit statistically significant) increase in ...."

Reviewer #3

The manuscript by Singh and Skibbens provides novel mechanistic insight into how cells monitor cohesin dysfunction by showing that wild-type Mcd1 is actively degraded through a surveillance pathway involving San1 and Das1 E3 ubiquitin ligases, revealing a previously unappreciated genome integrity safeguard with implications for cohesinopathies and cancer. The work is experimentally rigorous, combining genetics, biochemistry, and imaging to directly test hypotheses, rule out alternatives, and demonstrate rescue of cohesin mutant phenotypes through Mcd1 overexpression. The data strongly support the conclusion that active degradation of Mcd1, rather than passive destabilization, drives cohesin mutant lethality, though clarifying San1/Das1's direct role in proteasomal targeting would further strengthen the case. The manuscript is well-written, logically organized, and supported by robust quantification, with only minor typographical issues. By linking cohesin quality control to broader genome stability and disease contexts, this study offers significant and original contributions that make it a good candidate for publication in PLOS Genetics. However, this reviewer has some major concerns which needs to be addressed.

We thank the reviewer for this concise and supportive summary of our work.

Major Comments

(a) Direct evidence of Mcd1 proteolysis: To firmly establish that Mcd1 is actively degraded (and not just diluted or mislocalized), the authors should consider adding a direct assay for Mcd1 stability. For example, treating cells with a proteasome inhibitor during the S-phase arrest could show whether Mcd1 levels are rescued when proteolysis is blocked. Alternatively, examining ubiquitination of Mcd1 (via immunoprecipitation of Mcd1 and blotting for ubiquitin) in wild-type vs. cohesin-mutant cells would be very informative. Currently, the conclusion of proteasome-mediated degradation rests on genetic evidence (involvement of E3 ligases), which is strong but could be complemented by in vitro biochemical validation including the E2 enzyme.

We acknowledge that there are alternate explanations for the effects observed beyond Mcd1 degradation - although the reduction in Mcd1 is both impressive and ubiquitous across the cohesin alleles tested. To more directly test a degradation model, we exploited the well-established proteosome inhibitor MG-132. As the reviewer is likely aware, MG-132 entry and retention in yeast cells is effective only in a couple of circumstances. Our initial attempts included pre-treatment of cells with SDS (3 hours incubation prior to lysis). Unfortunately, we were unable to recover any appreciable levels of Mcd1. We note that cohesin mutations produce cell wall defects (Kothiwal et

al., 2021; Cena et al., 2007), such that SDS treatment resulted in a adverse effect that included Mcd1 reduction.

We then turned to the second strategy - deletion of the efflux pump, Pdr5, which otherwise pumps MG-132 out of cells (Gallen A collins, 2010). Following the deletion of PDR5 gene, log phase eco1Δ rad61Δ pdr5Δ triple mutant cells were arrested in HU for 2 hours, followed by addition of DMSO (solvent control) or MG-132 for 2 hours. Remarkably, changes in Mcd1 protein levels remained similar across cells incubated in DMSO or MG-132 during S phase (Fig S3 B,C), suggesting that Mcd1 degradation occurred elsewhere in the cell cycle. Since Mcd1 is also essential during G2 and M phases, but minimally produced during G1 phase (Guacci, 1997), we assessed the extent to which Mcd1 degradation occurs prior to anaphase onset. Log phase cultures of eco1Δ rad61Δ pdr5Δ cells were incubated in media that contains nocodazole (to synchronize cells preanaphase) along with either MG-132 (proteosome inhibitor) or a DMSO control. The progression of cells from log-phase growth to a pre-anaphase arrest was monitored by flow cytometry (Fig 6A). Extracts of the resulting cultures were then assessed by Western blot (Fig 6B). Quantification of the resulting blots reveal that cells incubated in the presence of MG-132 contain significantly elevated (nearly 3 fold) levels of Mcd1 compared to DMSO control (Fig 6C). Together, these results reveal that degradation occurs prior to anaphase onset, when Mcd1 is essential for sister chromatid cohesion, by the proteosome. The extent to which Mcd1 is similarly degraded in G1 or G2 phases remains for future studies.

(b) Mcd1 levels in suppressor mutants: As noted above, a crucial control would be to measure Mcd1 protein in the eco1Δ rad61Δ san1Δ and eco1Δ rad61Δ das1Δ strains. The manuscript shows that deleting these ligases improves growth, implying Mcd1 is preserved; actually demonstrating Mcd1 restoration in these backgrounds (via Western blot) would directly tie the suppression to Mcd1 stability.

*We thank the reviewer for recommending this experiment, which was also raised by Reviewer 1. As addressed above (see responses to Reviewer 1), eco1Δ rad61Δ cells and 3 biological replicates of the eco1Δ rad61Δ san1Δ triple mutant cells were arrested in S phase (see Fig A, above). Following cell lysis, Mcd1 and Pgk1 (loading control) protein levels were assessed by Western blot. Quantification of Mcd1 protein levels, normalized to Pgk1, revealed that SAN1 deletion elevated Mcd1 protein levels by ≈35% in eco1Δ rad61Δ cells. (see Figs B and C). Statistical significance was assessed using a one sample t test, two tailed, **, $P \leq 0.01$.*

(c) Redundancy of San1 and Das1: Since San1 and Das1 each partially alleviate the phenotype, the authors might explore whether deleting both together has an additive effect (i.e., a san1Δ das1Δ double in the eco1Δ rad61Δ background). This could establish whether these ligases act in the same pathway or have complementary roles in targeting Mcd1. If constructing that strain is not feasible, the authors should at least discuss the possibility of redundant or sequential action by these E3s. San1 typically targets misfolded proteins for degradation;

therefore, the observed reduction of Mcd1 may reflect preferential elimination of its misfolded form, a distinction that should be explicitly delineated.

We also wondered whether co-deletion of SAN1 and DAS1 would synergistically promote eco1Δ rad61Δ growth at elevated temperatures by increasing Mcd1 levels. We first assessed whether co-deletion of SAN1 and DAS1 impacts wildtype cell viability (Fig A, shown below). A streak assay comparing wildtype (YPH499) and three independent isolates of san1Δ das1Δ (YBS4869, YBS4870, YBS4871) cells revealed no detectable effect on cell viability at either at 30°C or 37°C (Fig A, below). Since wildtype cells withstand the absence of these two E3 ligases, we crossed san1Δ das1Δ cells to the eco1Δ rad61Δ cells and dissected the resulting tetrads to obtain the eco1Δ rad61Δ san1Δ das1Δ quadruple mutant cells. Log phase cultures of wildtype (YPH499), eco1Δ rad61Δ san1Δ (YGS290) and eco1Δ rad61Δ san1Δ das1Δ (YGS370, YGS371) were serially diluted onto rich medium plates and incubated at 30°C or 37°C (Fig. B, shown below). Our results revealed no additional suppression of temperature sensitivity in eco1Δ rad61Δ san1Δ das1Δ cells beyond that provided by SAN1 deletion alone. Future efforts will determine whether San1 and Das1 target the same ubiquitination site of Mcd1, or instead whether the contribution of Das1 is simply masked by the stronger effect by San1.

A

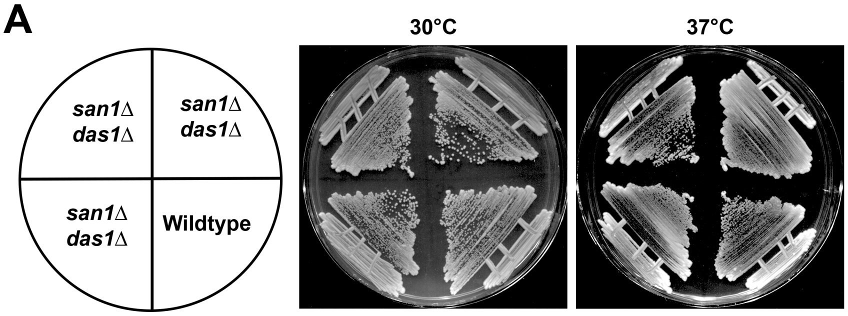

B

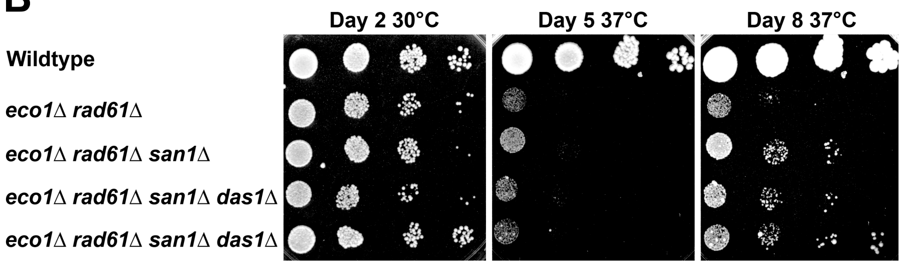

(d) Upstream signals: The discussion mentions Cln2-CDK as a potential upstream signal for this pathway. If possible, testing a cln2Δ mutant for suppression of Mcd1 loss (or cohesin mutant growth defects) would directly support that idea. However, this may be beyond the scope of the current work; at minimum, the authors should clarify that the Cln2 connection, while intriguing, remains speculative in this study.

We are equally excited the role that CDK regulation plays in cohesin pathways. While the mechanism may be speculative, we point out several recents studies that document that CLN2 deletion rescues both eco1Δ rad61Δ and pds5Δ elg61Δ growth defects/inviability (Buskirk and Skibbens, 2023; Choudhary et al., 2023; Singh and Skibbens, 2024). Moreover, the effect of CLN2 deletion occurs by increasing Mcd1 levels (above references). It would be interesting to test the extent to which CLN2 deletion also rescues mcd1-1 mutant cell phenotypes as a first step in linking CDKs to cohesin regulation, but given that this current study is focused on novel Mcd1 degradation pathways, we agree with the reviewer that this is outside our current scope.

(e) Rad21 amplification and elevated protein levels are observed in a significant subset of human tumors, particularly epithelial cancers. A discussion of how cancer cells override the normal proteolytic mechanisms that regulate Rad21/Mcd1 stability would help underscore the broader implications of the current findings.

An excellent point and we appreciate the opportunity to explore this topic. The manuscript has been modified by the following addition:

"Finally, Mcd1 targeting appears to extend beyond cells that exhibit cohesin defects. For instance, exposing cells to reactive oxygen species also triggers Mcd1 degradation via an apoptotic pathway (118–121). The conserved nature of a surveillance mechanism that targets Mcd1 is supported by findings that RAD21 (homolog of Mcd1) is degraded by caspases 3 and 7 during apoptotic responses (118, 122, 123). Together, these results suggest that inactivating cohesin functions through Mcd1/RAD21 degradation (through E3 ligases or caspases) may represent an evolutionarily conserved mechanism for promoting cell death. Conversely, cells that evade this surveillance system may obtain proliferative benefits. In support of this model, elevated expression of almost every cohesin subunit (RAD21, SMC1A, SMC3, STAG1, PDS5, WAPL) and cohesin regulator (NIPBL, MAU2) appears to be oncogenic and present in a wide range of cancer cells (44, 45). In fact, 30 of 33 different cancer types were recently shown to express elevated levels of ESCO2 (46). RAD21, however, is the most frequently amplified cohesin in non-small-cell lung cancer, breast cancer cells, cervical cancer, ovarian cancer, oral cancer and prostate cancer (40, 124–131). How cancer cells circumvent the transcriptional and degradational mechanisms that normally regulate RAD21 levels remains unclear, but reducing RAD21 levels in cancer cells appears effective in reducing cell proliferation and/or inducing apoptosis (124, 125, 130, 131). These findings, in combination with our current study, highlight the potential for targeting Mcd1, the master regulator of cohesin function, as a novel therapeutic strategy across multiple cancer types."

I consider (a) and (b) to be important controls to include to solidify the central claim of proteolytic Mcd1 turnover.

Minor:

1. Ensure all acronyms (e.g., "ts" for temperature-sensitive) are defined on first use for clarity.

Thanks - these have been corrected

2. Use a consistent format for yeast gene and protein names. For example, RAD61 (gene) vs. Rad61 (protein) and the deletion allele (rad61Δ) should be clearly distinguished. In the manuscript, “rad61D” appears in place of “rad61Δ” – this should be corrected to the standard delta symbol to avoid confusion.

Thanks - these have been corrected and standardized nomenclatures adopted.

3. There are minor typos in some captions. In Figure 3, the caption begins with a duplicated panel label (“A) ... A) wildtype”), which should be corrected. Double-check all figure labels and references in the text to ensure they match (e.g., if the text refers to Figure 2A, that it indeed corresponds correctly).

Thank you - the text has been revised as described.

4. There is a duplicated reference number in the text (ref. 95 appears twice in a row, page 12, line 255), which likely needs fixing in the bibliography. Ensure all references cited (especially in the introduction/discussion) are included and up-to-date. For example, references supporting the role of Wapl/Rad61 and Eco1 are cited; adding a brief citation or footnote in the introduction for San1 and Das1 (to note their known functions) might be helpful for readers.

Good catch. References and explanations of activities are now included in the revised manuscript for Rad61, Eco1, San1, etc. We also corrected the error in our reference list (and citation in text). We chose to introduce E3 ligases in the Results, only after we provided evidence that negated a role for Esp1 in Mcd1 degradation.

5. There are some minor typos (for example, eco1D rad61D is sometimes written without the Δ symbol in the Results, and pds5Δ elg1Δ appears as pds5D elg1D in one instance).

Thanks for pointing out these issues - we corrected the text.

Again - thank you all for your time and attention. We feel that, in responding to your comments, that this version of the study is more clear, more impactful and will be of significant benefit to researchers of cohesin biology and human maladies.

Gurvir and Bob
